# Linked Intronic Polymorphisms of the *PNPLA3* Gene Are Associated with Serum Markers of Liver Injury in Patients with Spontaneous HCV Clearance

**DOI:** 10.3390/ijms27010473

**Published:** 2026-01-02

**Authors:** Karina Gonzalez-Aldaco, Luis A. Torres-Reyes, Arturo Panduro, Sonia Roman

**Affiliations:** 1 Centro Universitario de los Valles, Universidad de Guadalajara, Carretera Guadalajara-Ameca Km. 45.5, Ameca 46600, Jalisco, Mexico; luis.torres9386@academicos.udg.mx; 2 Department of Genomic Medicine in Hepatology, Civil Hospital of Guadalajara, “Fray Antonio Alcalde”, Hospital #278, Col. El Retiro, Guadalajara 44280, Jalisco, Mexico; apanduro53@gmail.com (A.P.); soniamariaroman@hotmail.com (S.R.); 3 Health Sciences Center, University of Guadalajara, Guadalajara 44340, Jalisco, Mexico

**Keywords:** aminotransferases, platelets, APRI, liver damage, chronic HCV

## Abstract

Genetic variation in *PNPLA3* influences liver fat accumulation and hepatocellular injury in various liver diseases. However, the role of *PNPLA3* intronic polymorphisms in hepatic damage among hepatitis C virus (HCV) patients remains unclear. This study aims to investigate the association of three intronic *PNPLA3* polymorphisms (rs4823173, rs2896019, and rs2281135) with liver injury in HCV-infected patients with spontaneous HCV clearance (SC) and chronic hepatitis C (CHC). A total of 218 HCV-positive individuals were classified into SC (n = 64) or CHC (n = 154) groups. *PNPLA3* genotypes were determined by qPCR using TaqMan probes and liver damage through serum markers, noninvasive index, and liver stiffness. Among SC patients, the genotypes AA-rs4823173, GG-rs2896019, and AA-rs2281135 were associated with higher AST, ALT, and APRI, as well as decreased platelet counts, compared with patients homozygous for the non-risk genotypes (*p* < 0.05). No associations were found in CHC patients. The three polymorphisms were in perfect linkage disequilibrium (r^2^ = 1). The risk haplotype AGA was associated with higher AST and ALT, as well as lower platelet counts (*p* < 0.05) in SC patients. *PNPLA3* intronic polymorphisms and their association with serum liver injury markers could help identify hepatic injury in HCV-negative patients.

## 1. Introduction

Hepatitis C virus (HCV) is a hepatotropic virus that remains a leading cause of chronic liver disease worldwide [1]. Chronic HCV infection (CHC) continues to account for a substantial global health burden, including approximately 242,000 deaths and 1.5 million new infections annually [2]. CHC develops in around 75% of people who acquire HCV. Viral persistence can induce hepatic steatosis, fibrosis, cirrhosis, and hepatocellular carcinoma (HCC) [3]. Moreover, spontaneous clearance (SC) in the acute phase (<6 months) occurs in a variable percentage of patients (20–40%) [4]. Factors affecting SC are host, immune, viral, environmental, and genetic factors [5]. Patients who spontaneously cleared the infection may still progress in liver injury, highlighting the role of host and genetic factors in modulating hepatic outcomes [6].

Obesity is a significant risk factor for the development of metabolic dysfunction-associated steatotic liver disease (MASLD). However, individual susceptibility to MASLD varies considerably by ethnicity [7]. MASLD has rapidly become the most common cause of liver disease worldwide, currently affecting 38% of the global population. Although MASLD does not always progress to advanced liver disease, it has become the leading cause of liver transplantation [8]. Several factors, including environmental, metabolic, immune, genetic, and epigenetic factors, can affect the progression of MASLD to severe forms of the disease [9].

Genetic factors play a crucial role in the onset and progression of steatotic liver disease (SLD). The Patatin-like phospholipase domain-containing protein 3 (*PNPLA3*) gene, which encodes a triglyceride hydrolase, is among the most strongly associated genes with liver damage and SLD worldwide [10,11]. The genetic influence of *PNPLA3* on the risk of MASLD-related liver injury becomes progressively stronger with increasing body mass index (BMI) [12]. Evidence from Hispanic pediatric populations shows that *PNPLA3* variants are associated with increased hepatic fat accumulation and metabolic alterations, underscoring the BMI-dependent nature of this genetic risk [13]. Due to genetic predisposition and high prevalence of overweight and obesity in the Mexican population [14], it is crucial to explore the effect of *PNPLA3* polymorphisms on liver damage. Among HCV patients, the *PNPLA3* gene has been shown to contribute to HCC development, elevated alanine aminotransferase levels, and faster progression of cirrhosis among patients infected with genotype 1b [15]. However, the contribution of *PNPLA3* variations in HCV infection remains largely unknown. A Genome-Wide Association Study (GWAS) of Mexican Americans identified three single-nucleotide polymorphisms (SNPs) in the *PNPLA3* gene that were associated with elevated liver enzyme levels in a cohort of subjects with various metabolic and cardiometabolic diseases [16]. These SNPs are intronic variants, and their protein effects remain unknown. This study aimed to assess the contribution of the *PNPLA3* polymorphisms rs4823173 (G>A), rs2896019 (T>G), and rs2281135 (G>A) to liver damage in patients with HCV infection.

## 2. Results

### 2.1. Participant Characteristics

The demographic and clinical characteristics of 64 SC and 154 CHC patients are given in Table 1. The average ages of SC and CHC patients were 44.7 ± 13.0 years and 50.7 ± 11.8 years, respectively (*p* = 0.001). No significant differences were found in gender, BMI, and glucose between the two groups. Triglyceride, low-density lipoprotein cholesterol (LDL-c), very-low-density lipoprotein cholesterol (VLDL-c), total cholesterol, and platelet counts were higher in SC patients than in CHC patients (*p* < 0.001). Conversely, levels of insulin, aspartate aminotransferase (AST), alanine aminotransferase (ALT), gamma-glutamyl aminotransferase (GGT), AST-to-platelet ratio index (APRI), liver stiffness, and Fibrosis-4 index (FIB-4) were significantly higher in CHC patients than in SC patients (*p* < 0.05).

### 2.2. Genetic Information

The basic information on the three SNPs located in the *PNPLA3* gene (rs4823173, rs2896019, and rs2281135), along with their allelic frequencies in SC and CHC patients, is presented in Table 2. No differences were found in the Minor Allele Frequency (MAF) among SC and CHC patients.

### 2.3. Genotypic Frequency of PNPLA3 Polymorphisms

The genotyping frequency of polymorphisms in the *PNPLA3* gene is shown in Table 3. The three SNPs were in Hardy–Weinberg Equilibrium (HWE) in both patient groups (*p* > 0.05). Among SC patients, the rs4823173 SNP showed a GA heterozygote prevalence of 56.3%, followed by GG homozygotes at 23.4% and AA homozygotes at 20.3%. The same pattern was observed in rs2896019 and rs2281135, with TG-56.3%, TT-23.4%, and GG-20.3% and GA-56.3%, GG-23.4%, and AA-20.3%, respectively. Among CHC patients, the rs4823173 SNP showed a GA heterozygote prevalence of 50.6%, followed by GG homozygotes at 29.2% and AA homozygotes at 20.1%. The same pattern frequency was observed in rs2896019 and rs2281135 with TG-50.0%, TT-29.2%, and GG-20.8% and GA-50.0%, GG-29.2%, and AA-20.8%, respectively. We did not find an association between *PNPLA3* polymorphisms and the outcome of HCV infection.

### 2.4. Effect of PNPLA3 Polymorphisms on Serum Liver Damage Markers and Noninvasive Liver Damage Indices in HCV Patients

When each SNP was analyzed within groups separately, we found that, in patients with SC, the rs4823173 AA genotype was significantly associated with higher AST levels than the GG genotype (*p* = 0.042). The AA genotype was also associated with higher ALT and APRI when compared with the GG genotype (*p* = 0.016) and (*p* = 0.038), respectively, as well as with lower platelet counts compared with the GG genotype (*p* = 0.035). Same associations were observed for rs2896019 and rs2281135. The rs2896019 GG genotype and the rs2281135 AA genotype were both associated with increased AST, ALT, and APRI levels, as well as reduced platelet counts (Table 4). In contrast, no significant associations were detected between genotypes and clinical parameters in patients with chronic infection (Table 5).

### 2.5. Linkage Disequilibrium and Haplotype Analysis

**Figure 1:**
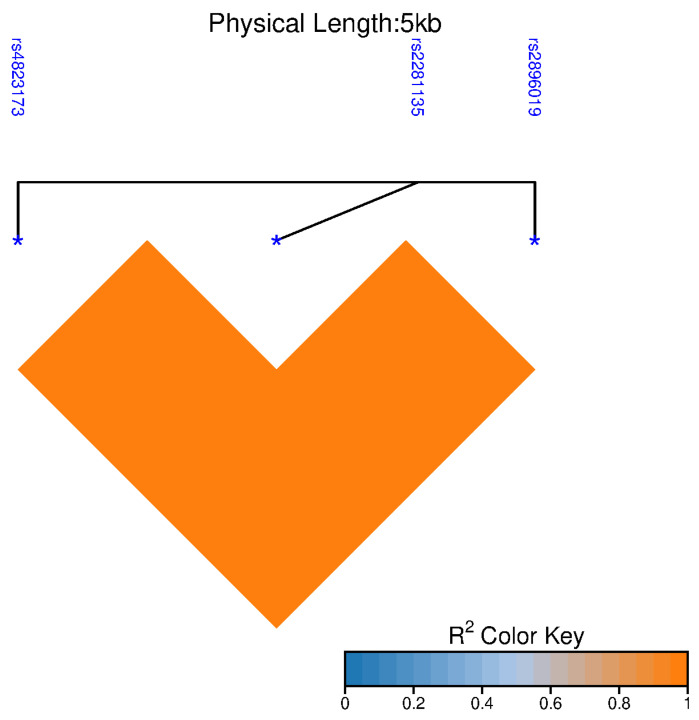
Linkage disequilibrium heatmap showing pairwise r^2^ values among the three intronic *PNPLA3* polymorphisms.

Given the proximity of the three SNPs, linkage disequilibrium (LD) analysis was performed (Figure 1). The three SNPs showed positive LD (*p* < 0.05), with a strong correlation (r^2^ = 1). Haplotype analysis revealed two major haplotypes across the study groups (Table 6). The haplotype (rs4823173/rs2896019/rs2281135) carrying the protective alleles (GTG) was the most frequent, present in 51.6% of SC and 54.2% of CHC patients. Conversely, the haplotype containing the risk alleles (AGA) was detected in 48.4% of SC and 45.8% of CHC patients, with no significant difference in distribution between the groups.

### 2.6. Effect of PNPLA3 Haplotypes on Serum Liver Damage Markers and Noninvasive Liver Damage Indices in HCV Patients

**Figure 2:**
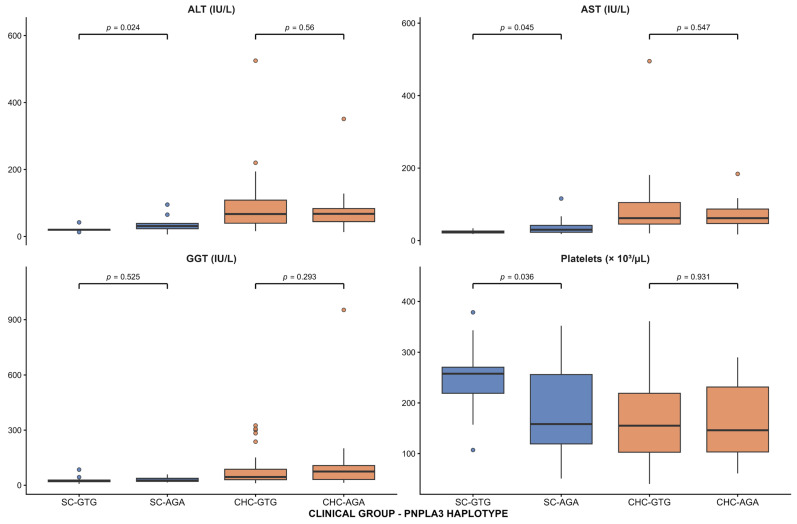
Association of *PNPLA3* haplotypes with serum liver damage markers in HCV patients. Box-and-whisker plots showing ALT, AST, GGT, and platelet count levels by *PNPLA3* haplotype (GTG and AGA) in patients with spontaneous clearance (SC) and chronic hepatitis C (CHC). Boxes represent the interquartile range (IQR), the central line indicates the median, and whiskers extend to 1.5 × IQR; points represent outliers.

**Figure 3:**
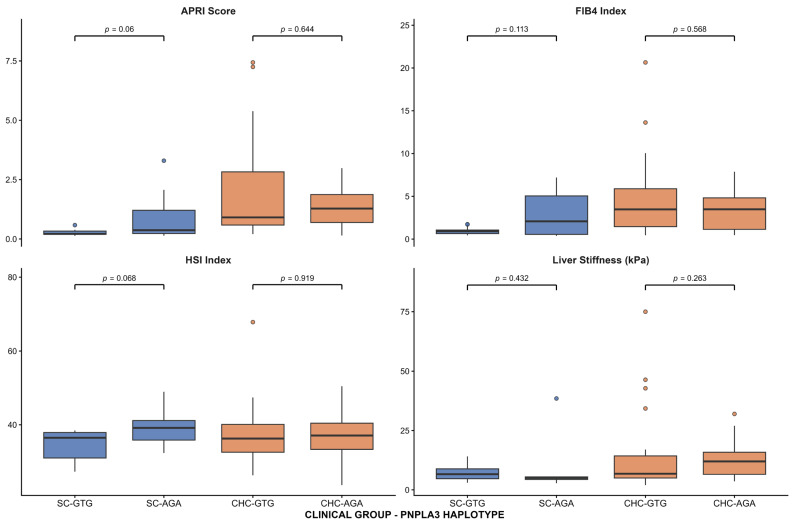
Association of *PNPLA3* haplotypes with noninvasive liver damage indices in HCV patients. Box-and-whisker plots showing APRI score, FIB-4, HIS, and liver stiffness according to *PNPLA3* haplotypes (GTG and AGA) in patients with spontaneous clearance (SC) and chronic hepatitis C (CHC). Boxes represent the interquartile range (IQR), the central line indicates the median, and whiskers extend to 1.5 × IQR; points represent outliers.

After determining haplotype frequencies, we evaluated the associations between the two *PNPLA3*-identified haplotypes and serum liver damage markers (AST, ALT, GGT, and platelet counts) and noninvasive liver damage indices (APRI levels, FIB-4, HIS, and liver stiffness) in SC and CHC patients. In SC group, the risk AGA haplotype was associated with higher levels of ALT (35.2 ± 23.2 vs. 20.9 ± 6.1 IU/L, *p* = 0.024) and AST (40.9 ± 27.4 vs. 24.2 ± 4.6 IU/L, *p* = 0.045), as well as lower levels of platelet counts (185.4 ± 88.0 vs. 247.6 ± 67.1 × 10^3^/µL, *p* = 0.036), compared with the GTG haplotype (Figure 2). No significant associations were found among noninvasive liver damage indices (Figure 3). Among the CHC group, no significant associations were observed between haplotypes and serum liver damage markers or noninvasive liver damage indices.

### 2.7. Impact of BMI on Liver Injury Markers Across PNPLA3 Haplotypes

**Figure 4:**
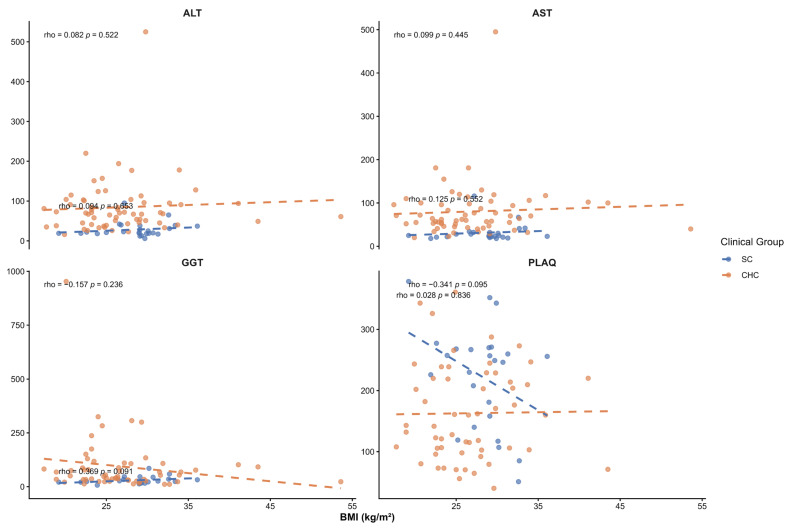
Spearman correlation between BMI and serum liver injury markers in SC and CHC patients. Correlation coefficients (ρ) and *p*-values are displayed in each panel.

**Figure 5:**
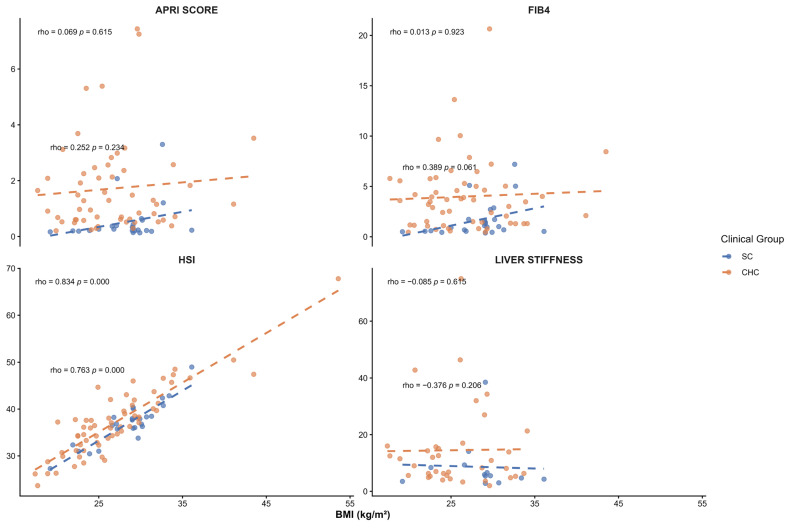
Spearman correlation analysis between BMI and noninvasive fibrosis indices in SC and CHC patients. Spearman’s ρ and *p*-values are indicated in each panel.

Given the known influence of BMI on liver enzyme levels and fibrosis-related markers, BMI was evaluated as a potential confounding factor in the association between *PNPLA3* haplotypes and liver injury. BMI was compared across *PNPLA3* haplotypes in both study groups, and no significant differences were observed within the SC or CHC groups (*p* > 0.05) (Table 7). In addition, no significant correlations were observed between BMI and serum liver injury markers in either group (Figure 4). We found a positive correlation with HSI (*p* < 0.05) as expected, in both groups, as BMI is included in its calculation (Figure 5). Overall, the association between *PNPLA3* haplotypes and serum liver injury markers in SC patients appears to be independent of BMI.

## 3. Discussion

In this study, we evaluated the effects of three intronic polymorphisms in the *PNPLA3* gene on liver damage, as assessed by serum markers, noninvasive scores, and transient elastography, in patients with HCV infection. We found an association between the three SNPs and elevated AST, ALT, and APRI levels, as well as reduced platelet counts, in patients who have cleared the infection, but not in those with CHC. These associations were also observed at the haplotype level. To the best of our knowledge, this is the first report describing this association in individuals with a history of HCV infection who achieved spontaneous viral clearance.

Over the past 15 years, *PNPLA3* has emerged as one of the most influential genetic determinants of liver disease [17]. To date, studies of *PNPLA3* gene polymorphisms have primarily focused on the rs738409 (I148M) variant and its association with MASLD [18]. Nonetheless, GWAS studies across populations have identified mutations in *PNPLA3* as contributing to steatotic liver disease and liver damage beyond the rs738409 variant [16,19,20]. In this context, increasing attention has been given to intronic variation within the *PNPLA3* locus as a potential contributor to interindividual and population-specific differences in liver disease susceptibility. Although these variants do not alter the protein-coding sequence, several studies have reported associations between intronic *PNPLA3* polymorphisms and liver-related phenotypes, suggesting that they may capture relevant regulatory or haplotypic effects influencing disease expression [21]. The evaluation of multiple intronic markers within the locus may therefore provide complementary information to coding variants, particularly in populations with complex genetic backgrounds, and help explain variability in liver injury severity that is not fully accounted for by rs738409 alone [22]. It was recently reported that intronic polymorphisms in *PNPLA3* (rs4823173, rs2896019, and rs2281135) are associated with elevated liver aminotransferases among Mexican American subjects with different metabolic and cardiometabolic diseases, as well as in Mexican patients with MASLD [16,23]. However, the effect of these variants on liver damage in Mexican patients with HCV infection remains unknown.

The Mexican population has a tripartite genetic background (Amerindian, Caucasian, and African), remarkably implicated with increased risk of diverse health issues, including liver diseases [24,25]. Recent lifestyle changes, particularly the westernization of diet, have been identified as a major contributor to this current risk pattern among the mestizo population [25]. Currently, approximately 75% of Mexicans are overweight or obese, and a high prevalence of MASH has been reported among young individuals with obesity [14,26]. Given this genetic and metabolic context, it is relevant to explore how specific genetic variants contribute to liver injury in this population.

In this study, the three SNPs in the *PNPLA3* gene—AA-rs4823173, GG-rs2896019, and AA-rs2281135—and their risk haplotype (AGA) were associated with elevated ALT and AST levels in patients who cleared HCV infection, but not in CHC patients, supporting a haplotype-dependent effect of *PNPLA3* variation in patients who cleared HCV infection. Information on the stage of liver fibrosis is crucial for managing patients with hepatitis C, as it provides prognostic information and assists in therapeutic decisions [27]. Although liver biopsy represents the gold standard for evaluating the stage of liver fibrosis, it remains an invasive procedure with inherent risks [28]. However, over the past decade, imaging techniques and serum marker tests have become key methods for assessing liver injury, often obviating the need for liver biopsy and histological analysis [29]. It is accepted that blood levels of ALT and AST are a consequence of liver cell membrane damage, but also reflect relevant aspects of liver physiology and pathophysiology beyond hepatocyte membrane disruption [30]. ALT and AST are usually elevated in chronic HCV infection in parallel with the severity of liver disease, which returns to the normal range after viral eradication [31,32]. Although in the group of SC patients, the means of ALT and AST were under the normal range, patients carrying the risk genotypes reached the highest levels of AST (>42 IU/L) and ALT (>37.2 IU/L) comparing with non-carries, indicating that patients with SC may be under the influence of other liver damage triggers or the aftermath of hepatitis C infection. Studies have shown that HCV infection results in persistent epigenetic and transcriptional changes associated with fibrosis and HCC, suggesting that viral cure only partially eliminates the virus-induced pro-fibrogenic and carcinogenic effects [33,34]. These results highlight the need for clinical follow-up of patients with SC or sustained virological response to monitor liver damage risk. Moreover, the loss of association in the CHC group may be explained by the predominant influence of chronic inflammation, fibrosis progression, and viral and host factors that affect aminotransferase levels, which could overshadow the effect of the genetic variation [6]. These results are in line with studies conducted on Japanese and Pakistani chronic HCV-infected patients, where no correlation was found between the rs738409 variant and liver fibrosis [35,36]. The divergence between both groups suggests that *PNPLA3*-related metabolic and inflammatory pathways may exert a greater effect under conditions of limited viral injury or after viral clearance. Additional studies using human-centric models or humanized mouse models will be required to identify the mechanisms underlying this process [37].

Although liver fat content and its association with *PNPLA3* genotypes were not assessed in this study, the SC group’s mean BMI was in the overweight range. To evaluate whether BMI could confound the observed associations, Spearman correlation analyses were performed between BMI and liver injury markers and noninvasive indices. No significant correlations were observed between BMI and serum liver enzymes (ALT, AST, GGT), platelet count, APRI, FBI-4, or liver stiffness. A positive correlation with the HSI was observed, as expected, since BMI is an integral component of its calculation. Overall, these findings suggest that the associations observed between *PNPLA3* haplotypes and liver injury markers in SC patients are not driven by BMI. A previous analysis of this group of patients reported a dietary pattern characterized by western-type foods, including pork, red meat, soft drinks, bacon, and fried foods [38]. Therefore, we cannot exclude the possibility that some patients presented with MASLD due to dietary factors or *PNPLA3* variants. Whether these patients have increased hepatic fat accumulation remains to be determined. Indeed, one of the main limitations of our study is the absence of a formal diagnosis of fatty liver. Observational studies have reported an increased likelihood of coronary and carotid atherosclerosis during HCV infection [39]. Given the dietary patterns among Mexican SC patients and the effect of *PNPLA3* variants on atherosclerosis development [40], these patients should be followed to monitor the development of cardiometabolic disease.

Platelets serve as an essential indicator of liver function in patients with chronic liver disease [41]. Thrombocytopenia results from decreased production of the hormone thrombopoietin (TPO) in the damaged liver and/or increased platelet destruction due to phagocytosis in an enlarged spleen. Additionally, impaired hematopoiesis in the bone marrow due to viral infection may further reduce platelet count [42]. A decreased peripheral platelet count may indicate a more advanced degree of fibrosis in hepatitis C [43]. Because platelets play a crucial role in liver regeneration, low platelet count exacerbates hepatocyte injury and promotes the progression of cirrhosis [44]. In the present study, the three *PNPLA3* risk genotypes, AA-rs4823173, GG-rs2896019, and AA-rs2281135, and the risk haplotype AGA were associated with lower platelet counts among SC patients. This finding is in accord with the described effect of the *PNPLA3* at-risk alleles on platelet count [45]. The *PNPLA3* rs738409 variant has been identified as a modifier of platelet count by exome-chip meta-analysis [46]. In MASLD patients, the expression of genes involved in platelet biogenesis was associated with *PNPLA3* GG at rs738409 [45], supporting the concept that *PNPLA3* variants are associated with platelet count. However, the impact of *PNPLA3* on platelets is still largely unexplored.

In accordance with the association between elevated aminotransferases and low platelet count, the three risk genotypes were also associated with APRI levels. APRI is a noninvasive index based on AST, ALT, and platelet counts that predicts fibrosis and cirrhosis in HCV patients [47]. Interestingly, no significant associations were observed between *PNPLA3* genotypes or haplotypes and FIB-4, HSI, or liver stiffness. This apparent discrepancy may reflect differences in the biological processes captured by these indices. APRI is primarily driven by aminotransferase levels and platelet count, and is particularly sensitive to early or mild hepatic injury [48]. In contrast, FIB-4 and liver stiffness are more closely related to advanced fibrosis and structural liver changes [49,50], which may be less prevalent in patients with spontaneous HCV clearance. Similarly, HSI incorporates metabolic components such as BMI and diabetes, and therefore may not accurately reflect genetically driven liver injury in this context [51]. These findings suggest that *PNPLA3* intronic variants may preferentially influence biochemical and hematological markers of liver injury rather than established fibrotic remodeling. Thus, determining *PNPLA3* at-risk genotypes could help predict which patients who reach SC or SVR are at risk of developing liver damage.

Genomic and personalized medicine require consideration of the regional genetic and cultural background [52]. In this context, integrating genetic information with clinical and biochemical markers could improve the early detection of individuals at risk of MASLD or liver injury, enabling tailored preventive and therapeutic approaches [53]. Emerging evidence suggests that modulation of PNPLA3-related pathways may enable personalized strategies to prevent progression of liver injury, particularly in genetically susceptible individuals [54]. The present findings emphasize the importance of incorporating genomic profiling into clinical practice to better account for interindividual variability in liver disease susceptibility and progression.

## 4. Materials and Methods

### 4.1. Study Subjects

A total of 218 unrelated, treatment-naïve adults with positive anti-HCV antigen were consecutively enrolled between January 2014 and December 2016 at the Department of Molecular Biology in Medicine at the Hospital Civil de Guadalajara “Fray Antonio Alcalde” (Guadalajara, Jalisco, Mexico). Individuals coinfected with the hepatitis B virus or human immunodeficiency virus, autoimmune disease, Child–Pugh class B or C, Wilson’s disease, hemochromatosis, excessive alcohol intake (men, >30 g/day; women, >20 g/day), and use of hypolipidemic drugs were excluded. Informed written consent was obtained from each patient, and the study protocol was reviewed and approved by the Institutional Ethics Committee (CI-06018). The study complied with the ethical guidelines of the 2013 Declaration of Helsinki.

### 4.2. Clinical Evaluation

Clinical records were compiled by a physician and included demographic information, clinical data, risk factors for HCV acquisition, and laboratory test results. Serological screening for anti-HCV antibodies was performed using a third-generation ELISA (AxSYM^®^, Abbott Laboratories, Abbott Park, IL, USA). Quantitative detection of HCV RNA in serum was carried out with a standardized real-time PCR assay (Roche COBAS AmpliPrep/COBAS TaqMan 48 HCV test, Pleasanton, CA, USA).

Based on virological criteria, patients were classified into two groups. The SC group (n = 64) consisted of individuals with at least two undetectable HCV RNA results within the preceding 12 months, separated by an interval of at least six months. The CHC group (n = 154) included patients with two detectable HCV RNA results within the same timeframe and interval. None of the patients had received a prior diagnosis or treatment for HCV infection at the time of enrollment.

### 4.3. Anthropometric Assessment

Body mass index (kg/m^2^) was estimated using electrical bioimpedance (InBody3.0, Analyzer Body Composition, Biospace, Seoul, South Korea). Normal weight was defined as >18.5–24.99 kg/m^2^, overweight as >25–29.99 kg/m^2^, and obesity as >30 kg/m^2^ according to the WHO classification [55].

### 4.4. Biochemical Measurements

Blood samples were drawn after an 8 h fast. Biochemical measurements of AST, ALT, GGT, platelets, triglycerides, total cholesterol, and glucose were performed using a Vitros 250 analyzer (Ortho-Clinical Diagnostic, Johnson & Johnson, Rochester, NY, USA). Commercial control serum and human pooled serum were used to ensure the accuracy of the biochemical measurements. LDL-c concentration was calculated using the Friedewald formula [56], and VLDL-c concentration was calculated as TC-(LDL-c + HDL-c). Fasting insulin levels were measured by an enzyme-linked immunosorbent assay (Monobind Inc., Lake Forest, CA, USA).

### 4.5. Liver Stiffness and Fibrosis Measurements

Liver stiffness was evaluated by a certified physician using transient elastography (FibroScan^®^, Echosens, Paris, France). Results were expressed in kilopascals (kPa) and reported as the median value of ten valid measurements. Liver fibrosis was further assessed using noninvasive indices, APRI, calculated according to the formula: APRI = AST [IU/L]/upper limit of normal [IU/L] × 100/Platelet count (10^9^/L) [47]. FIB-4, calculated as: (age × AST) ÷ (platelet count × (sqr(ALT)) [50] and HIS = 8 × (ALT/AST ratio) + BMI (+2, if female; +2, if diabetes mellitus) [57].

### 4.6. Genotyping

Genomic DNA was extracted from peripheral whole-blood leukocytes using the salting-out method and stored at −80 °C until use. The genotypes of the *PNPLA3* polymorphisms were determined using a 5′ allelic discrimination method. A qPCR using TaqMan^®^ SNP Genotyping Assays was carried out (rs4823173 C_25931728_10, rs2896019 C_1840500_10, rs2281135, C_15875080_10, Applied Biosystems, Foster, CA, USA). Cycling conditions were as follows: an initial enzyme activation step for 10 min at 95 °C, followed by 40 cycles of denaturation for 15 s at 95 °C and annealing/extension for 1 min at 60 °C, using a StepOnePlus Thermocycler (Applied Biosystems, Foster, CA, USA). Genotype calling was verified using positive and negative controls. Twenty percent of the samples were genotyped in duplicate, and 100% concordance was observed. 

### 4.7. Statistical Analysis

Statistical analyses were performed using IBM SPSS Statistics version 19.0 for Windows (IBM Corp., Armonk, NY, USA) and R version 4.5.0 [58]. Categorical variables were expressed as frequencies and compared using the chi-square or Fisher’s exact test. For descriptive statistics, continuous variables were expressed as mean ± standard deviation. Quantitative data were compared using one-way analysis of variance (ANOVA), the Kruskal–Wallis test, or the *t*-test or Mann–Whitney U test, as appropriate. Post hoc *t*-tests were conducted as needed to assess intergroup differences, depending on the homogeneity of variances (Tukey’s test for normally distributed variables with homogeneity of variances, or Dunn’s test with a Bonferroni correction for nonparametric variables). A *p*-value < 0.05 (two-tailed) was considered statistically significant.

Genotypic and allelic frequencies of *PNPLA3* polymorphisms were obtained by the direct counting method. HWE and haplotype inference were performed using Arlequin v3.5.2.1 for Windows [59]. LD (r^2^) was calculated by the Genetic Data Analysis (GDA) program (version 1.0) [60].

## 5. Conclusions

Our results suggest that *PNPLA3* intronic polymorphisms are associated with serum markers of hepatic injury in patients with spontaneous HCV clearance but not in those with chronic infection. These findings support the role of host genetic factors in modulating liver outcomes beyond active viral replication and underscore the utility of noninvasive biomarkers for detecting liver injury. Future studies with larger cohorts and mechanistic approaches are needed to confirm these associations and clarify their clinical implications. It has been documented that, after viral eradication, patients are already at risk of developing advanced liver damage complications [61]. How to treat and manage these patients is a new challenge faced by hepatologists. The use of polymorphisms in the *PNPLA3* gene and their correlation with serum markers of liver injury can help diagnose liver injury in HCV-negative patients.

## Figures and Tables

**Table 1:** Demographic and clinical characteristics of HCV-infected patients.

Variable	Spontaneous Clearance	Chronic Infection	*p*-Value
Number of subjects	64	154	
Age (years)	44.7 ± 13.0	50.7 ± 11.8	0.001
Male n (%)	31 (48.4)	61 (39.6)	0.293
Female n (%)	33 (51.6)	93 (60.4)	
BMI (kg/m^2^)	27.8 ± 3.8	27.08 ± 5.5	0.085
Fasting glucose (mg/dL)	102.1 ± 36.4	104.9 ± 45.5	0.367
Insulin (µlU/mL)	12.3 ± 15.2	15.2 ± 13.3	0.033
Triglycerides (mg/dL)	172.6 ± 84.1	125.4 ± 54.8	<0.001
LDL-c (mg/dL)	114.0 ± 87.9	87.9 ± 37.3	<0.001
VLDL-c (mg/dL)	33.8 ± 16.4	25.0 ± 10.9	<0.001
Total cholesterol (mg/dL)	186.7 ± 44.3	151.5 ± 43.0	<0.001
AST (IU/L)	31.2 ± 18.0	76.0 ± 57.0	<0.001
ALT (IU/L)	31.9 ± 21.1	79.4 ± 71.4	<0.001
GGT (IU/L)	41.5 ± 71.5	78.8 ± 103.9	0.005
Platelets × 10^3^/µL	221.9 ± 79.9	158.3 ± 90.3	<0.001
Liver stiffness (kPa)	11.6 ± 16.0	15.2 ± 14.1	0.007
APRI	0.4 ± 0.5	3.5 ± 19.7	<0.001
FIB-4	1.5 ± 1.43	9.4 ± 57.7	<0.0001
HSI	37.7 ± 6.4	36.9 ± 7.0	0.359

**Table 2:** Basic information on the three SNPs of the *PNPLA3* gene.

dbSNP	Gene	Chr	Base Pair	Location	Allele	MAF		*p*-Value
						SC	CHC	
rs4823173	*PNPLA3*	22	43,932,850	Intron variant	G/A	0.484	0.455	0.643
rs2896019	*PNPLA3*	22	43,937,814	Intron variant	T/G	0.484	0.458	0.688
rs2281135	*PNPLA3*	22	43,936,690	Intron variant	G/A	0.484	0.458	0.688

Chr: chromosome; SC: spontaneous HCV clearance; CHC: chronic hepatitis C; MAF: minor allele frequency.

**Table 3:** Genotypic frequencies of *PNPLA3* polymorphisms in HCV patients.

dbSNP	Spontaneous Clearance	Chronic HCV	*p*-Value
rs4823173			
GG	15 (23.4)	45 (29.2)	0.481
GA	36 (56.3)	78 (50.6)	0.545
AA	13 (20.3)	31 (20.1)	1.000
HWE	0.451	1.00	
rs2896019			
TT	15 (23.4)	45 (29.2)	0.481
TG	36 (56.3)	77 (50.0)	0.488
GG	13 (20.3)	32 (20.8)	1.000
HWE	0.451	1.00	
rs2281135			
GG	15 (23.4)	45 (29.2)	0.481
GA	36 (56.3)	77 (50.0)	0.488
AA	13 (20.3)	32 (20.8)	1.000
HWE	0.453	1.00	

HWE: Hardy–Weinberg equilibrium.

**Table 4:** Association of *PNPLA3* gene polymorphisms with liver damage markers and noninvasive liver damage indices in SC patients.

dbSNP	Genotype	n	AST, IU/L	ALT, IU/L	GGT, IU/L	Platelets, ×10^3^/µL	APRI Score	Liver Stiffness	FIB-4	HSI
rs4823173	GG	15	24.3 ± 4.7	20.8 ± 6.3	28.6 ± 20.1	247.4 ± 67.2	0.3 ± 0.1	7.2 ± 3.8	0.9 ± 0.4	35.0 ± 3.7
	GA	36	30.3 ± 16.1	35.0 ± 23.1	49.8 ± 92.4	229.6 ± 81.0	0.4 ± 0.2	12.8 ± 18.0	1.3 ± 0.8	38.3 ± 7.6
	AA	13	42.7 ± 27.9	37.2 ± 23.2	31.7 ± 13.5	171.5 ± 75.7	0.9 ± 1.0	11.1 ± 15.3	3.0 ± 2.4	39.3 ± 4.7
	*p*-value		0.042 ^a^	0.016 ^b^	0.644	0.035 ^d^	0.038 ^c^	0.723	0.070	0.245
rs2896019	TT	15	24.3 ± 4.7	20.8 ± 6.3	28.6 ± 20.1	247.4 ± 67.2	0.3 ± 0.1	7.2 ± 3.8	0.9 ± 0.4	35.0 ± 3.7
	TG	36	30.3 ± 16.1	35.0 ± 23.1	49.8 ± 92.4	229.6 ± 81.0	0.4 ± 0.2	12.8 ± 18.0	1.3 ± 0.8	38.3 ± 7.6
	GG	13	42.7 ± 27.9	37.2 ± 23.2	31.7 ± 13.5	171.5 ± 75.7	0.9 ± 1.0	11.1 ± 15.3	3.0 ± 2.4	39.3 ± 4.7
	*p*-value		0.042 ^a^	0.016 ^b^	0.644	0.035 ^d^	0.038 ^c^	0.723	0.070	0.245
rs2281135	GG	15	24.3 ± 4.7	20.8 ± 6.3	28.6 ± 20.1	247.4 ± 67.2	0.3 ± 0.1	7.2 ± 3.8	0.9 ± 0.4	35.0 ± 3.7
	GA	36	30.3 ± 16.1	35.0 ± 23.1	49.8 ± 92.4	229.6 ± 81.0	0.4 ± 0.2	12.8 ± 18.0	1.3 ± 0.8	38.3 ± 7.6
	AA	13	42.7 ± 27.9	37.2 ± 23.2	31.7 ± 13.5	171.5 ± 75.7	0.9 ± 1.0	11.1 ± 15.3	3.0 ± 2.4	39.3 ± 4.7
	*p*-value		0.042 ^a^	0.016 ^b^	0.644	0.035 ^d^	0.038 ^c^	0.723	0.070	0.245

Values are expressed as mean ± standard deviation. Serum liver injury markers include AST, ALT, GGT, and platelet count. Noninvasive indices include APRI, FIB-4, HSI, and liver stiffness. ANOVA and Kruskal–Wallis tests were used to calculate *p*-values based on normality and homoscedasticity. rs4823173: ^a,b,c^, and ^d^ AA vs. GG, *p* < 0.05. rs2896019: ^a,b,c^, and ^d^ GG vs. TT, *p* < 0.05. rs2281135: ^a,b,c^, and ^d^ AA vs. GG, *p* < 0.05.

**Table 5:** Association of *PNPLA3* gene polymorphisms with liver damage markers and noninvasive liver damage indices in CHC patients.

dbSNP	Genotype	n	AST, IU/L	ALT, IU/L	GGT, IU/L	Platelets, ×10^3^/µL	APRI Score	Liver Stiffness *	FIB-4	HSI
rs4823173	GG	45	85.1 ± 75.7	90.3 ± 84.5	78.5 ± 86.4	164.1 ± 84.1	1.9 ± 1.9	14.3 ± 17.32	4.3 ± 4.1	37.3 ± 7.5
	GA	78	73.7 ± 51.2	74.8 ± 67.9	69.5 ± 73.5	153.1 ± 99.5	5.3 ± 27.1	16.7 ± 13.6	14.3 ± 79.2	36.7 ± 6.8
	AA	31	69.5 ± 34.6	75.4 ± 59.2	105.3 ± 176.4	164.2 ± 73.6	1.3 ± 0.8	13.1 ± 8.2	3.3 ± 2.2	37.2 ± 7.2
	*p*-value		0.519	0.274	0.381	0.523	0.817	0.661	0.391	0.863
rs2896019	TT	45	85.1 ± 75.7	90.3 ± 84.5	78.5 ± 86.4	164.1 ± 84.1	1.9 ± 1.9	14.3 ± 17.3	4.3 ± 4.1	37.3 ± 7.5
	TG	77	74.1 ± 51.4	75.4 ± 68.1	68.7 ± 73.7	153.7 ± 100.1	5.3 ± 27.3	16.7 ± 13.6	14.4 ± 79.7	36.8 ± 6.8
	GG	32	68.5 ± 34.4	73.8 ± 58.8	106.2 ± 173.2	162.1 ± 73.1	1.3 ± 0.8	13.6 ± 8.2	3.3 ± 2.1	36.8 ± 7.2
	*p*-value		0.566	0.344	0.262	0.591	0.784	0.661	0.422	0.968
rs2281135	GG	45	85.1 ± 75.7	90.3 ± 84.5	78.5 ± 86.4	164.1 ± 84.1	1.9 ± 1.9	14.3 ± 17.3	4.3 ± 4.1	37.3 ± 7.5
	GA	77	74.1 ± 51.4	75.4 ± 68.1	68.7 ± 73.7	153.7 ± 100.1	5.3 ± 27.3	16.7 ± 13.6	14.4 ± 79.7	36.8 ± 6.8
	AA	32	68.5 ± 34.4	73.8 ± 58.8	106.2 ± 173.2	162.1 ± 73.1	1.3 ± 0.8	13.6 ± 8.2	3.3 ± 2.1	36.8 ± 7.2
	*p*-value		0.566	0.344	0.262	0.591	0.784	0.661	0.422	0.968

Values are expressed as mean ± standard deviation. Serum liver injury markers include AST, ALT, GGT, and platelet count. Noninvasive indices include APRI, FIB-4, HSI, and liver stiffness. ANOVA and Kruskal–Wallis tests were used to calculate *p*-values based on normality and homoscedasticity. * Liver stiffness analyses include only subjects with available measurements.

**Table 6:** Haplotype frequencies of *PNPLA3* gene polymorphisms in SC and CHC patients.

dbSNP	Haplotype	SC	CHC	*p*-Value
rs4823173/rs2896019/ rs2281135	GTG	51.6	54.2	0.800
	AGA	48.4	45.8	

**Table 7:** Comparison of BMI according to *PNPLA3* haplotypes in SC and CHC patients.

Clinical Group	Haplotype	BMI	*p*-Value
SC	GTG	27.2 ± 3.6	0.182
	AGA	29.4 ± 3.7	
CHC	GTG	27.1 ± 6.2	0.508
	AGA	26.0 ± 5.8	

Values are expressed as mean ± standard deviation. *p*-values were calculated using the Wilcoxon rank-sum test within each clinical group. BMI: Body mass index; SC: spontaneous clearance; CHC: chronic HCV infection.

## Data Availability

All datasets generated for this study are included in the article. Further inquiries can be directed to the corresponding author.

## Abbreviations

The following abbreviations are used in this manuscript:

*PNPLA3*	Patatin-like phospholipase domain-containing protein 3
HCV	Hepatitis C virus
CHC	Chronic HCV infection
SC	Spontaneous HCV clearance
LD	Linkage disequilibrium
HCC	Hepatocellular carcinoma
SNP	Single-nucleotide polymorphism
AST	Aspartate aminotransferase
ALT	Alanine aminotransferase
APRI	AST-to-platelet ratio index
FIB-4	Fibrosis-4
HSI	Hepatic steatosis index
MASLD	Metabolic dysfunction-associated steatotic liver disease
SLD	Steatotic liver disease
GWAS	Genome-Wide Association Study
HWE	Hardy–Weinberg Equilibrium
